# Family Perceptions of Barriers and Facilitators of a Telehealth Program to Support Infants at Risk for Developmental Delays

**DOI:** 10.34763/jmotherandchild.20252901.d-24-00042

**Published:** 2025-07-02

**Authors:** Kathlen Terezinha Montes Soares Fernandes, Ana Luiza Righetto Greco, Nayara Rodrigues Gomes de Oliveira, Maja Medeiros, Alicia Spittle, Cibelle Kayenne Martins Roberto Formiga

**Affiliations:** Department of Physiotherapy, Universidade Estadual de Goiás, Goiânia, GO, Brazil; Hospital das Clínicas da Universidade Federal de Goiás, Goiânia, GO, Brazil; Faculty of Medicine, Dentistry and Health Sciences, University of Melbourne

**Keywords:** Child development, early intervention, remote consultation, eHealth, telemedicine

## Abstract

**Background:**

Telehealth was an alternative in many countries during the COVID-19 pandemic for infants at risk of developmental delays. However, some barriers still challenge the adoption of telehealth as a care option, particularly once face-to-face interventions recommenced. This study aimed to identify the barriers and facilitators of a telehealth program to support infants at risk for developmental delays.

**Materials and methods:**

A prospective longitudinal study was conducted with 30 infants born at risk of developmental delay (preterm or term, with mean age of 3.1 months). Infants were enrolled between 2–12 months of corrected age. The program consisted of weekly telehealth sessions with a physical therapist focusing on supporting children’s cognitive, motor, speech, and language development. After 6 months, the caregivers answered a questionnaire on perceived barriers and facilitators of the telehealth program.

**Results:**

A mean of 9.5 (range 2–12) sessions were carried out. Most caregivers (80%) felt comfortable and satisfied with the program, found the application for video calls easy to use, got help with their questions, and perceived improvements in the development of their infants. The main barrier was most caregivers rated the concern regarding their child as low (53.3%).

**Conclusion:**

Caregivers considered the telehealth program satisfactory and viable for complementary care and monitoring of infants’ development.

## 1. Introduction

Challenges faced during the COVID-19 pandemic included the severity of cases and the need to ensure continuity of care for different populations [1]. The social isolation interrupted health services, leaving families and their infants without face-to-face assistance. In this scenario, telehealth was an alternative to different countries [2].

Telehealth integrates information and communication technologies into remote activities and has become increasingly frequent [3]. Specifically, it allows the remote monitoring of patients who need continuous care without traveling and transportation costs [4]. Although this modality has already been used in several countries (the United States, Canada, Japan, China, and Australia), it was regularized and implemented in Brazil only during the pandemic [5].

Studies have shown several advantages of telehealth for assisting children with disabilities (e.g., low-cost access to health information, flexible schedules, the possibility of intervention in the home environment, and active participation by caregivers), which resulted in a better perception of their development by caregivers [5–9]. The facilitators of telehealth also extend to infants at high risk of cerebral palsy. Schlitching et al. (2022) [10] assessed the effects of a telerehabilitation program performed five times a week for 12 weeks during the pandemic in infants at high risk of cerebral palsy (aged between 3 and 18 months). The program included activities for motor development, environmental enrichment, guidance on properly positioning the infant, and educational strategies for caregivers. According to the results, most infants’ motor development improved, demonstrating the efficacy of the program.

Despite these facilitators, some barriers still challenge the adoption of telehealth as a care option, including lack of internet access, unavailability of schedules in the family routine, and additional responsibilities imposed on caregivers [6,10]. Since most studies were conducted during the pandemic, it is essential to understand whether the facilitators and barriers of telehealth remain after the pandemic to assess the feasibility of telehealth as an alternative option for infant care. In this sense, the study aimed to identify the barriers and facilitators of a telehealth program to support infants at risk for developmental delays.

## 2. Materials and methods

### 2.1 Study design

This longitudinal prospective study was approved by the human research ethics committee of the State of University of Goias and the Hospital das Clínicas of the Federal University of Goias (protocol number CAAE 42042820.8.0000.8113), Brazil. The study followed the Brazilian national ethical principles and the Declaration of Helsinki. Informed consent was obtained from all subjects involved in the study.

### 2.2 Participants

The sample for this study consisted of thirty infants and their caregivers selected through convenience sampling. Participants were recruited from a follow-up program composed of a multidisciplinary team, including physical therapists and pediatricians of Hospital das Clinicas of Universidade Federal de Goias between August 2022 and January 2023. The following inclusion criteria were applied: admission to the neonatal intermediate care unit after birth, participation in a developmental follow-up program, and a corrected age of between 2 and 12 months at enrollment.

The G*Power software (version 3.2.1) was used to calculate the sample size, and a total of 30 infants was found based on an effect size of 0.50, an α value of 5%, and a statistical power of 0.85, recommended to minimize type I error. Considering the at-risk infants admitted to the follow-up program between 2022 and 2023 (n = 138), 66 were under 12 months of corrected age.

### 2.3 General procedures

#### 2.3.1 Medical records

Data were collected from medical records and from the physical therapists at the follow-up clinic. These include gestational age, weight, and length at birth and Apgar score at the first and fifth minutes.

#### 2.3.2 Face-to-face consultation

At the first face-to-face appointment, caregivers were informed about the study and invited to participate. Gross motor development was measured by the Alberta Infant Motor Scale (AIMS) according to the instruments’ recommendations [11]. The socioeconomic status of the family was estimated using the Brazilian Economic Classification Criterion [12], which classifies families from A (best economic status) to D or E (worst economic status). Maternal educational level and family income were also recorded to analyze the epidemiological and socioeconomic profiles of family members and infants.

### 2.4 Telehealth program

Those who showed interest received a message via the mobile app WhatsApp containing the objectives, the routine of appointments, the duration of sessions, and a total number of sessions. The physical therapist scheduled the sessions according to the family’s availability and routines, facilitating the accessibility of the telehealth. The program consisted of 20-minute synchronous video calls in the home environment once a week. Video calls aimed to guide the development of the infant by proposing specific exercises to support cognitive, motor, speech, and language development skills in the family context.

At the beginning of the first session, the physical therapist spoke with the caregiver and observed the home environment to identify possible physical barriers and facilitators for implementing the telehealth program. Also, guidance was provided regarding the ideal positioning of the infant and the mobile device, the use of appropriate clothing to avoid restricting the infant’s movements, the importance of having a toy or object to attract the infant’s attention during the session, and the most appropriate infant behavioral states for the sessions. This initial assessment and orientation were fundamental to ensure the effectiveness and productivity of subsequent sessions.

After observing the environment and interacting with the caregiver, the physical therapist assessed the infant’s development using the Ages and Stages Questionnaire—third edition (ASQ-3) to identify the infant’s specific development level, establish the objectives of the telehealth program according to the identified needs of the infant, and provide guidance to parents. The ASQ-3 is a tool adapted for the Brazilian population to screen the developmental progress of children with a corrected age of 1 to 60 months [13]. The questionnaire has 30 questions and five domains that address the development of communication, gross motor, fine motor, problem-solving, and personal-social aspects. Responses are scored as follows: “yes” (10 points), “sometimes” (5 points), and “not yet” (0 points). Domain scores are classified into three categories: “does not require assessment,” “requires additional activities with monitoring,” or “requires in-depth professional assessment” [14]. The main advantage of using it in the study was that the ASQ-3 covers different areas of the infant’s development, and helped ensure that the guidance provided to parents was not limited to just motor development milestones. The results from the ASQ-3 were used to inform the therapist’s individualized approach for each session, ensuring that the objectives set for each infant were aligned with their developmental progress. This initial assessment with AIMS and ASQ-3 enable the therapist to tailor the telehealth program according to the infant’s developmental specific needs, based on their individual development and the family’s environment.

When necessary, the caregiver received verbal and visual guidance on adequate postures for the infant during the assessment. At the end of the first session, the physical therapist identified specific areas that needed additional support.

In the second session, the objectives were defined with the caregiver, and the proposed activities were adapted according to the family routine. The activities were established individually, based on the AIMS and ASQ-3 assessments along with typical development expected for the age.

Examples of the activities included instructions on how to position the infant prone in case of difficulty keeping the head in the midline and turning sideways. The caregiver was instructed to position a rolled-up towel under the chest of the infant (armpit level) to provide support for the upper torso, facilitate head elevation and cervical rotation, and improve comfort and safety. Other instructions included bringing hands to the midline, playing with the hands, reaching for and manipulating objects, bringing the hands to the feet, bringing the feet to the mouth, and training to roll over and sit with hands supported near the feet. For all activities, the caregiver was instructed to interact with the infant and motivate the active involvement using rattles, attractive objects, or verbal cues.

The physical therapist demonstrated the activities using an articulated doll and provided detailed instructions regarding the aim of the activity, posture, and response expected. The caregiver was then asked to practice the activity with the infant and with guidance provided when necessary. The physical therapist remained available to answer questions, offer feedback, and adjust the environment or posture of the infant.

Other than the sessions, all caregivers were instructed to practice the exercises twice a day without supervision of the physical therapist; the duration of each activity varied according to the availability of the caregiver and behavior and acceptance of the infant to the activity. During the activities, the infant had to be awake, active, and without signs of crying or discomfort. If the infant cried, the caregiver was advised to pause the activity and resume it once the infant was calmer and more receptive.

During the sessions, new objectives were set for caregivers if the infant had already achieved previous objectives. At the end of each session, the physical therapist filled in the diary with the date, start time, duration of the session, and the prescribed guidance.

After the last session, the caregivers were asked to complete a Google Forms questionnaire regarding their perceptions of facilitators and barriers to the telehealth program. Data were analyzed by an independent researcher blinded to the study.

#### 2.4.1 Diary telehealth program

A diary was created and utilized as a tool to characterize the telehealth program. The diary documented information about the telehealth program, including the city of origin of the family, the family member responsible for conducting the sessions, the number and duration of sessions (in minutes), and the total duration of the program (in months). The telehealth program ended in December 2023.

**Figure 1.**
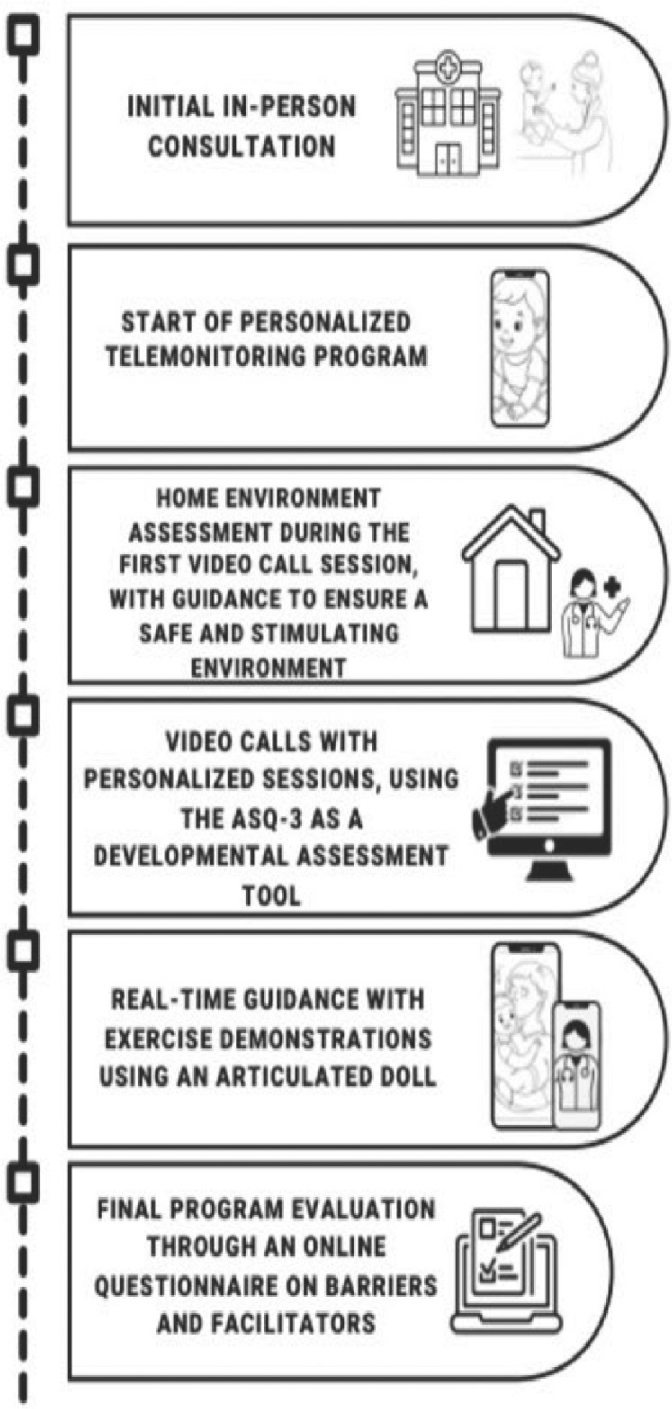
Steps of the telehealth program.

To visualize the steps of the procedure, see Figure 1, which presents a graphical representation of the implementation of the telehealth program.

### 2.5 Outcomes measurements

A self-administered questionnaire was designed to assess the perception of caregivers regarding facilitators and barriers during the telehealth program. The language was adapted according to the educational level of the caregiver to avoid technical terms. The questionnaire had 22 affirmative sentences, including the following: “I felt comfortable taking video calls with the physical therapist”; “The video calls were tiring for me”; “I had difficulty understanding the instructions given by the physical therapist”; “I felt more confident in carrying out the stimulation exercises with my infant”; and “The telehealth program helped improve the development of my infant”.

The responses were collected using a 5-point Likert scale: “strongly disagree”, “disagree”, “neither agree nor disagree”, “agree”, and “strongly agree”. To calculate the scores, responses were assigned numerical values: “strongly disagree” was equivalent to “1”, “disagree” to “2”, “neither agree nor disagree” to “3”, “agree” to “4”, and “strongly agree” to “5”. Statements related to the infant telehealth program were classified as facilitators (positive aspects, such as perceived effectiveness and ease of use) or barriers (negative aspects, such as perceived technical difficulties and challenges in implementing guidance). For instance, statements like “I found it easy to use the video call app” or “I thought it was a great idea to use video calls as a complement to in-person appointments” were classified as facilitators, as they reflect positive caregiver perceptions telehealth program. Conversely, statements such as “I had trouble finding a suitable location in my home for video calls” or “I felt overwhelmed with stimulating the child at home” were considered barriers, as they express unfavorable experiences. A question about the experience with the telehealth program was also added (scores ranged from 0 to 10).

### 2.6 Statistical analysis

Data were presented as mean (standard deviation) for continuous variables and relative frequency (percentage) for categorical variables. The Shapiro-Wilk test verified the normality of the data distribution. For the characterization of the telehealth program, descriptive statistics were used to summarize the caregivers who conducted the sessions, the number of sessions, the duration of sessions, and the total program duration. For the analysis of responses from the self-administered questionnaire on facilitators and barriers, descriptive statistics were used to summarize the caregiver perceptions of the telehealth program. The Statistical Package for the Social Sciences (version 19, IBM Corp, USA) was used in the analysis.

## 3. Results

Thirty infants participated and completed the program. Table 1 shows characteristics of infants and sociodemographic data of the family.

**Table 1.** Sample characterization

**Characteristics (n = 30)**	**Values**
Sex - f (%)	
Female	15 (50)
Male	15 (50)
Gestational age (weeks) - M (SD)	
Apgar score in the fifth minute - M (SD)	8.0 (± 1.6)
Birth weight (g) - M (SD)	1961.6 (± 711.6)
Chronological age before the program (months) - M (SD)	3.1 (± 1.1)
Corrected age before the program (months) - M (SD)	1.6 (± 0.9)
AIMS Score * (First consultation) – f (%)	
Atypical	18 (60)
Suspicious	9 (30)
Normal	3 (10)
ASQ-3 (Before the program) – f (%)	
Communication (Normal/Requires follow-up or professional evaluation)	20 (66.7) / 10 (33.4)
Gross Motor Coordination (Normal/Requires follow-up or professional evaluation)	8 (26.7) / 22 (73.4)
Fine Motor Coordination (Normal/Requires follow-up or professional evaluation)	20 (66.7) / 10 (33.3)
Problem-Solving (Normal/Requires follow-up or professional evaluation)	20 (66.7) / 10 (33.3)
Personal Aspects (Normal/Requires follow-up or professional evaluation)	16 (53.3) / 14 (46.7)
Maternal age - M (SD)	30.2 (± 6.1)
Socioeconomic level - f (%)	
B1	2 (6.7)
B2	8 (26.7)
C1	12 (40)
C2	7 (23.3)
D and E	1 (3.1)
Maternal educational level - f (%)	
Incomplete elementary school	1 (3.3)
Incomplete high school	5 (16.7)
Complete high school	14 (46.7)
Incomplete higher education	6 (20)
Complete higher education	4 (13.3)
Family income (Brazilian real)** - M (SD)	2+B3:C29924.67 (± 1446.39)

F = frequency; % = percentage; M = mean; SD = standard deviation; g = grams;

Total score by Alberta Infant Motor Scale (AIMS)*;

Md = median; min = minimum value; max = maximum value;

Brazilian Real** = monetary value used in Brazil.

Most caregivers present during the telehealth program sessions were mothers (86.7%). The families took a mean of 6 (± 3.2) months to complete the program and attended 9.5 (± 4.0) sessions, which lasted approximately 24.1 (± 4.98) minutes each (Table 2).

**Table 2.** Characterization of the telehealth program

**Variables (n = 30)**	**Values**
Caregiver that conducted the session - f (%)	
Mother	26 (86.7)
Father	2 (6.7)
Grandmother	1 (3.3)
Aunt	1 (3.3)
City of origin - f (%)	
Goiânia	7 (23.3)
Countryside of the state of Goiás	21 (70)
Another state	2 (6.7)
Time to complete the program (months) - M (SD)	6.3 (± 3.2)
minimum – maximum	1 – 12
Total number of sessions - M (SD)	9.5 (± 4.0)
minimum – maximum	2 – 12
Duration of the session (minutes) - M (SD)	24.1 (± 4.9)
minimum – maximum	15 – 32

f = frequency; % = percentage; M = mean; SD = standard deviation.

Out of the 30 babies who fully participated in the telemonitoring program, we received responses from 24 caregivers to the questionnaire on the perception of facilitators and barriers to the telemonitoring program, which was sent to them during the final appointment. Although it is not possible to determine with certainty the reasons for the non-response of the remaining six caregivers, it is likely that factors such as lack of time, perceived as unnecessary after the completion of telemonitoring, or individual needs contributed to this non-response. The research team made several attempts to ask the family to respond to the questionnaire, but we were unsuccessful.

Regarding facilitators, 80% felt comfortable participating, found the application for video calls easy to use, got help with questions, were satisfied with the service, and reported an improvement in the development of the infant. In addition, more than 70% agreed with the duration of sessions, felt confident to perform the stimuli with the infant, agreed with the compatibility of schedules for the session, found it easy to understand the physical therapist, reported that video calls were good to complement the infant care, and were satisfied with the program.

The following barriers were found: internet connection, difficulties finding an appropriate location for sessions, overload to perform the stimulus, showing the face during video calls, concerns about infant development, and that the telehealth program could have been better. The telehealth program received an average score of 9.7 (Table 3).

**Table 3.** Perception of caregivers regarding facilitators, barriers, and experience telehealth program

**Perceptions of caregivers (n = 24)**	**Agreement level**				
	**Totally disagree (1)**	**Disagree (2)**	**Neither agree nor disagree (3)**	**Agree (4)**	**Totally agree (5)**
**Barriers - n (%)**					
My internet hindered the video calls.	10 (33.3)	11 (36.7)	2 (6.7)	1 (3.3)	
My house is too busy for video calls.	8 (26.7)	16 (53.3)			
I had trouble finding a place to answer the video calls in my house.	7 (23.3)	16 (53.3)	1 (3.3)		
The video calls caused me shame and embarrassment.	14 (46.7)	10 (33.3)			
The video calls were tiring for me.	17 (56.7)	7 (23.3)			
I felt overwhelmed to stimulate the child at home.	13 (43.3)	7 (23.3)	4 (13.3)		
I needed help understanding the instructions given by the physical therapist.	11 (36.7)	13 (43.3)			
I felt insecure about showing my face or the face of the infant during the video calls.	11 (36.7)	11 (36.7)		2 (6.7)	
I care little about the development of my infant.	7 (23.3)	1 (3.3)		10 (33.3)	6 (20)
Telehealth program has not made any difference to the development of my infant.	15 (50)	9 (30)			
The telehealth program could have been better.	12 (40)	7 (23.3)	3 (10)	2 (6.7)	
**Facilitators - n (%)**					
I felt comfortable participating in the video calls with the physical therapist.				4 (13.3)	20 (66.7)
The app used for the video calls was easy to use.				10 (33.3)	14 (46.7)
The video calls were compatible with my schedule.			1 (3.3)	14 (46.7)	9 (30)
The duration of the video calls was sufficient to receive assistance from the physical therapist.		1 (3.3)	2 (6.7)	14 (46.7)	7 (23.3)
I found it easy to understand the physical therapist during the video calls.		2 (6.7)		9 (30)	13 (43.3)
Using video calls to complement the care was a great idea.			2 (6.7)	2 (6.7)	20 (66.7)
The video calls helped me clear up several doubts.				6 (20)	18 (60)
I felt more confident in carrying out the stimulation exercises with my infant.			3 (10)	5 (16.7)	16 (53.3)
I was satisfied with the physical therapist service.				5 (16.7)	19 (63.3)
I am very satisfied with the telehealth program.			2 (6.7)	6 (20)	16 (53.3)
The telehealth program helped improve the development of my infant.				10 (33.3)	14 (46.7)
**Experience with telehealth program** - mean (standard deviation)	9.71 (±0.46)				

app = application.

## 4. Discussion

This study applied a synchronous telehealth program to at-risk infants after the pandemic and assessed the perception of caregivers regarding the barriers and facilitators. The results showed a positive response to the program, in which the facilitators outweighed the barriers, indicating that the telehealth program can be a promising strategy.

Regarding the characteristics of the telehealth program, a higher participation of mothers was observed during sessions, which corroborates Provenzi et al. (2021) [15], who conducted telehealth appointments with children with neurological problems (mean age of 5.8 years). These findings highlight the central role of the mother as caregiver since she spends most of her time with the infant and performing chores.

Most infants and their caregivers lived far from the hospital where they were monitored face-to-face. These data corroborate the study by Juárez et al. (2018) [16] that highlighted the acceptance, feasibility, and accuracy of a teleassessment for the early identification of autism spectrum disorder in young children aged between 20 and 34 months. In addition, teleassessment sessions allowed saved, on average, three hours in travel time to specialized services [16]. Hatton et al. (2018) [17] also demonstrated that a telehealth program for auditory brainstem response was effective and reduced costs for caregivers facing geographical barriers. These findings highlight the importance of telehealth programs as a complementary tool to face-to-face monitoring in specialized services, especially for families facing difficulties accessing health services.

The follow-up during the telehealth program lasted longer than expected because of the behavior of infants (sleeping, crying, and irritability) on the day of the session, the daycare schedule, the availability of caregivers, and unforeseen events. Despite these challenges, the importance of communication during the program was highlighted since caregivers sought guidance from the physical therapist through messages, videos, and photos. Previous studies have shown the effectiveness of hybrid or asynchronous telehealth during the pandemic [5,8]. The asynchronous exchange of photos and videos may help with communication between caregivers and professionals, and alternating this modality with synchronous sessions may meet the preferences of caregivers [5,8]. Therefore, offering synchronous and asynchronous guidance can facilitate flexibility and be an alternative to the incompatibility of schedules.

Regarding the perception of caregivers, more facilitators than barriers were evidenced. The facilitators included comfort and satisfaction with the service, ease of use of the application for video calls, readiness of physical therapists to answer questions, and perception of improvement in infant development. Previous studies have highlighted the satisfaction and comfort of a telehealth program during the pandemic, reinforcing its potential [18–21] as an alternative type of care for at-risk infants.

Regarding barriers, most caregivers perceived they cared little for the development of their infants. In addition, the management of professional and family activities at the same time made the routine challenging. According to Schlichting et al. (2022) [10], the return to work hindered caregivers from continuing in a telerehabilitation program for at-risk infants conducted during the pandemic. Thus, the overload of responsibilities may lead to the perception of less time dedicated to the development of the infant [10]. These aspects should be considered when setting and adapting goals and activities during a telehealth program according to the family context.

The limitation of the study was the relatively small sample size, which may have hindered the generalization of the results to a broader context. The telehealth program requires the active participation of caregivers, which may result in dropouts over time, especially if the program extends beyond three months. Future studies with similar samples could benefit from adopting qualitative approaches to explore these perceptions more thoroughly and offer a more comprehensive understanding of the factors that influence participation in telehealth interventions.

The study revealed that the family had a positive perception of the telehealth program for high-risk infants after the pandemic. The facilitators overcame the barriers, indicating that telehealth may complement the pediatric physical therapy treatment, promote bonds between physical therapists and caregivers, provide individual guidance during the weekly monitoring, and reduce travel costs.

### Keypoints

- Facilitators outweighed the barriers, indicating that the telehealth program is a promising strategy.
- Telehealth enables active maternal participation.
- Telehealth was well-accepted by caregivers.
- Barriers included internet connectivity and space.
- Telehealth could be a viable strategy for supporting caregivers and monitoring infant development, particularly in low-resource settings where access to traditional healthcare services is limited.
